# “*Doctors are in the best position to know*…”: The perceived medicalization of contraceptive method choice in Ibadan and Kaduna, Nigeria

**DOI:** 10.1016/j.pec.2016.03.026

**Published:** 2016-08

**Authors:** Hilary M. Schwandt, Joanna Skinner, Abdulmumin Saad, Lisa Cobb

**Affiliations:** a Fairhaven College, Western Washington University, Bellingham, USA; b Center for Communication Programs, Johns Hopkins Bloomberg School of Public Health, Baltimore, USA; c Nigerian Urban Reproductive Health Initiative, Abuja, Nigeria

**Keywords:** Hormonal contraception, Service delivery, Family planning, Service provision, Nigeria, Urban

## Abstract

**Objectives:**

The medicalization and clinic-based distribution of contraceptive methods have been criticized as barriers to increasing levels of contraceptive use in Nigeria and other settings; however, our understanding of how clients themselves perceive the contraceptive method decision-making process is very limited.

**Methods:**

Focus group discussions among men and women in Ibadan and Kaduna, Nigeria, were used to examine attitudes and norms surrounding contraceptive method decision-making in September and October of 2010.

**Results:**

Choosing a family planning method was presented as a medical decision: best done by a doctor who conducts clinical tests on the client to determine the best, side effect free, contraceptive method for each client. An absolute trust in health professionals, hospitals, and governments to provide safe contraception was evident.

**Conclusion:**

The level of medicalization placed on contraceptive method choice by urban Nigerians is problematic, especially since a test that can determine what contraceptive methods will cause side effects in an individual does not exist, and side effects often do occur with contraceptive method use.

**Practice implications:**

Provider and client education approaches would help to improve client involvement in contraceptive decision-making and method choice.

## Highlights

• Contraceptive methods are selected by a doctor using clinical tests.
• Health professionals are trusted to provide risk-free contraception.
• Involving clients in contraceptive method choice will likely increase use.

## 1 Introduction

Nigeria is a country characterized by high fertility, high maternal mortality, and low contraceptive use. Only 10% of currently married Nigerian women are using modern methods—3% use injectables, 2% male condoms, 2% oral contraceptive pill, 1% IUD, 1% other, and less than 1% use implants and female sterilization [1]. In April of 2011 the federal government of Nigeria made contraceptives free in public health facilities [1]. Women can source contraceptives from a variety of public and private facilities in Nigeria; however, they most commonly use the private sector (60%), in contrast to the public sector (29%), or other options (11%) [1].

Considerable research has been conducted in developing countries, including Nigeria, to examine levels of contraceptive use and identify common barriers to family planning among married women, young women, and sexually active unmarried women [2], [3], [4]. Across West Africa, accessibility has been identified as a supply-side factor in the stagnantly low rates of contraceptive use in the region [2]. In Nigeria, demand side factors such as desire for more children, lack of knowledge, and opposition by the user or a family member are also barriers to contraceptive use [1], [5]. Fear of side effects and infertility are among the most common reasons for nonuse [6], [7] and are a concern for a variety of subpopulations in Nigeria, including university students [8], antenatal patients [5], and urban women [9].

Researchers have also identified common determinants of contraceptive method choice. Informed, appropriate, safe method choice is important for both uptake and continuation of family planning. Family planning programs that offer a balanced method mix allow for a wide variety of client preferences and method attributes [10]. Contraceptive method choice is one of the six elements of Bruce’s framework for quality of care in family planning and has been found to be an important determinant of contraceptive uptake and sustained use [11]. For example, in urban Pakistan, uptake of contraceptive methods was more likely in health centers that had more methods available [12], while in Indonesia clients who received their method of choice displayed the lowest rates of discontinuation at follow up [13]. The ability to switch methods is also important for long-term contraceptive use in order to meet the changing needs of clients depending on tolerance of side effects, stage of the life cycle, desired length of spacing, and other factors.

Bulatao developed a framework for understanding method choice based largely on evidence from Asia and the United States that emphasizes the individual and includes four dimensions, namely: contraceptive goals, contraceptive competence, contraceptive evaluation, and contraceptive access [14]. In these and other studies, client characteristics, such as: age, parity, education, exposure to family planning messages, and partner approval are known to influence method choice [15], [16], [17], [18]. Government policies and programs, history of method introduction and availability in the country, as well as method attributes such as cost, effectiveness, and ease of use are additional factors that have an effect on the method mix available to a population [10].

Evidence also exists of health facility barriers in method choice, including staff levels, expired stock, provider bias, and lack of training in Nigeria and other developing countries [3], [17], [18], [19]. The general medicalization of family planning and the clinic-based nature of distribution has been criticized as a barrier to increasing levels of contraceptive use [20], [21], [22]. These medical barriers include eligibility criteria, over applied contraindications, and numerous process hurdles that clients face when trying to obtain contraception [22]. For example, blood tests and pelvic exams may be administered before prescription of oral contraceptives, although these practices do not contribute substantially to safe and effective use of many contraceptive methods in the WHO Selected Practice Recommendations for Contraceptive Use [23]. In Nigeria, the National Family Planning/Reproductive Health Service Protocols [24] recommends the availability of certain screening tests, including urinalysis and blood tests, primarily to detect pregnancy and sexually transmitted infections, although these tests may not be commonly administered due to unavailability or cost.

Although many of these barriers have been identified in Nigeria, our understanding of how clients themselves perceive their choice of method is nonexistent. Who do they think has control over contraceptive method choice? How do clients perceive the process of deciding on a method?

This study uses qualitative data from the cities of Ibadan and Kaduna, Nigeria, to examine attitudes and norms surrounding contraceptive method choice decision-making. Understanding the client’s perspective on decision-making about choice of method has the potential to improve family planning programs and policies to increase initial uptake and sustained use of contraception.

## 2 Methodology

This study is part of a larger qualitative study that was designed to understand key factors influencing the demand for family planning in two urban areas of Nigeria. For this sub-study, focus group discussions were used to obtain information on contraceptive method decision-making, utilizing projective techniques, which provided an indirect approach to gain information about underlying norms that can be overlooked or otherwise influenced by direct questioning or facilitator bias.

### 2.1 Recruitment

Family planning service providers recruited individuals who were using family planning into the study at family planning facilities through the use of a screening questionnaire to determine eligibility.

To recruit individuals who had never used family planning a similar screening questionnaire was used at the community level with the assistance of community leaders who mobilized potential study participants. The community leaders were highly respected gate-keepers in their communities. The community leaders were first informed about the study purpose and objectives in order to get their support and approval prior to conducting the study in their communities. The community leaders were then asked to inform members of their communities about the study while emphasizing voluntary participation.

All potential study participants were initially approached, introduced to the study purpose and objectives, and asked if they were interested in participating in the study. Those who indicated an interest in participation were then screened for study participation. The screening questionnaire was structured to filter participants who did not meet the inclusion criteria with “yes” or “no” responses to questions directly connected to each inclusion criteria, which included: age (18–49) and residence.

Volunteers who agreed to participate were provided details about the study by the focus group discussion facilitators. All potential study participants who met the inclusion criteria and were interested in participation were asked for verbal consent prior to participation. Study participants were reassured of anonymity and confidentiality while obtaining consent.

### 2.2 Study sites

The study was conducted in two urban areas of Nigeria: Ibadan and Kaduna. Ibadan is located in south-western Nigeria and is the capital of Oyo State. Kaduna, the capital of Kaduna State, is located in north-western Nigeria. The two study sites were chosen for this study to represent the more conservative views of the north of Nigeria in contrast to the less conservative views of the southern region. The use of modern contraceptives in the south has exceeded that of the north. For example, Kaduna State had a modern contraceptive prevalence of 20% in 2013 as compared to 37% in Oyo State [1].

### 2.3 Study participants

Study participants were men and women of reproductive age who were residing in Ibadan and Kaduna, Nigeria. The focus group**s** were homogenous in respect to city, marital status, sex, age (18–24 years and 25–49 years), wealth, and family planning experience (for married women only). There was a total of 26 focus group discussions conducted in the two cities in September and October of 2010 (see Table 1). The focus groups ranged in size from 5 to 13 participants, with the average being 9 participants. A total of 243 individuals participated in the study—153 females and 90 males.

### 2.4 Topic guide

The topic guide was developed to use projective techniques to facilitate data collection on a sensitive topic in a group setting. A fictional short story was read aloud by the facilitators about a family with two young children—the youngest being just 1 and a half years old. In the story, the mother sees promotional materials for family planning at her local clinic and is interested in delaying her next birth. A few months after seeing the materials she returns to the clinic to discuss the topic of family planning. She is prescribed the oral contraceptive pill and starts to use the method. Following the story, the topic guide included semi-structured questions with follow-up prompts to guide the group discussion. The topic guide, including the fictional story, was the same for all focus groups.

The guide was translated into the local languages by a certified translator and back translated to English to assess accuracy of translation. It was pre-tested with urban residents during the facilitator training and further refined based on the pre-test results.

### 2.5 Facilitator training

Qualified and experienced research assistants native to and fluent in the local language were recruited and trained by the research firm hired to conduct the study. The training covered issues such as an overview of qualitative research methods, fieldwork ethics, and teamwork. The research teams were familiarized with the discussion guides in both English and the local languages; each question in the topic guide was thoroughly discussed. In addition, the research assistants carried out role-plays to practice leading focus group discussions.

### 2.6 Data collection

The focus group discussions were conducted by the trained facilitators. Sessions began with ice-breakers to build rapport between the facilitators and the participants. The focus group discussions were conducted in the local language and at a location within the community where the participants identified as conducive to discuss sensitive issues freely. A trained scribe took notes during the discussion, including non-verbal communication, while the other facilitator, the moderator, guided the discussion using the topic guide. Participants were served refreshments and provided with stipends to cover transportation costs. All discussions, with the consent of the participants, were audio taped and the recordings were transcribed verbatim in the local languages. The transcribed texts were then translated into English.

### 2.7 Analysis

Data sorting and analysis were carried out using ATLAS.ti software. Only one researcher coded the data. The data analysis was guided by the thematic content analysis approach [25]. In this study, ‘coding up’ as opposed to ‘coding down’ was utilized; the codes were developed based on the data and were not defined prior to data collection [26]. Using this approach, all transcripts were read multiple times to identify emerging themes and allow for the generation of the codes based upon the participants’ own words.

After all the transcripts were coded, a matrix was created to help identify patterns within the codes. Each row of the matrix was a focus group and the columns were the codes. The cells were then populated by the direct quotes from the study participants from each focus group for each code. The matrix was useful in grouping the different nuances within each theme, discerning differences and similarities between groups within themes, and making connections broadly between themes.

### 2.8 Ethical considerations

Ethical approval to conduct the study was obtained from the Institutional Review Board at the Johns Hopkins Bloomberg School of Public Health in Baltimore, Maryland, and at the Obafemi Awolowo University, Ile Ife, Nigeria. Additional approvals were obtained from the state Ministry of Health in the two states where the study was conducted.

Participation in all aspects of the study, in the screening, consenting, and discussions, were voluntary. Reminders about the option to withdraw and halt study participation were made throughout the recruitment, consent, and data collection processes.

## 3 Results

During the focus group discussions on contraceptive method decision-making four main themes emerged: (1) the importance of body system compatibility with the contraceptive; (2) doctors are the primary contraceptive decision-makers; (3) doctors use clinical tests to assess a client’s body system in order to identify the most compatible contraceptive; and (4) an absolute trust placed in health professionals, the health system, and the government in protecting the health of Nigerians through selecting, providing, and prescribing safe contraceptives. While these themes emerged in the discussions, not all participants in all focus group discussions contributed to the discussions. It is important to note that in the absence of direct contributions to these themes there was no counter-narrative provided.

### 3.1 Body system compatibility with contraceptives

Choosing a family planning method was presented as a medical decision. Study participants often mentioned the issue of whether a family planning method is compatible with a woman’s “body system” or “chemistry”: *One can*’*t say one method is better than this other one; it all depends on the body chemistry of each individual that partakes of it (*Female, 27 years, married, 1 child, family planning user, low SES, Ibadan). If a particular method is not compatible with the woman’s body system then she would suffer side effects from use of that method. *I think it (using injectables) is somewhat risky because our human bodies are different. What is good for one can be bad for another* (Male, 46 years, married, 3 children, middle SES, Kaduna).

Respondents were aware that different methods may be suitable for different people but this was linked strongly to the issue of “body chemistry” rather than fertility goals, actual experience of side effects, or method contraindications: *I think there is no straight answer, as long as it*’*s conducive with the user*’*s system, it is good. Different types are suitable for different people (*Female, 30 years, married, 0 children, family planning nonuser, low SES, Ibadan).

### 3.2 Doctors are the primary contraceptive decision-maker

Perhaps because of the perception that method choice is based on innate biological factors, health providers were identified as the primary decision-maker for choosing the appropriate family planning method for a woman: *Doctors are in the best position to know whether the pill is a good contraceptive method or not* (Male, 22 years, married, 1 child, middle SES, Ibadan). *A qualified doctor is in best position to decide* (Female, 22 years, married, 1 child, nonuser, middle SES, Ibadan). *It depends on doctor*’*s recommendation. If it is recommended by a medical doctor, then she can use it* (Male, 28 years, married, 1 child, middle SES, Ibadan).

It should be noted here that participants used the term “Doctor” to most likely refer to all health professionals, anyone with health-related training who works in a health facility, irrespective of whether they have a medical degree or not.

### 3.3 Doctors conduct clinical tests to determine body system type

Respondents described the decision-making process as dependent on clinical tests, which determine her body system type and the contraceptive method most suitable: *The method to use depends on the doctor*’*s prescription after the appropriate test has been conducted (*Male, 24 years, married, 5 children, middle SES, Ibadan). Such tests are perceived to predict whether or not a method is “good” for a particular individual, as explained by a married woman from Ibadan: *[Health professionals] normally conduct some tests and recommend the most befitting for a person so if it wasn*’*t good for her, it wouldn*’*t have been recommended for her (*35 years, 0 children, family planning nonuser, low SES). They also predict the presence or absence of side effects: *It is somewhat risky because some people use it (family planning) without conducting appropriate test and once they start using it, they begin to have complications. It is necessary and important for anyone who wants to use IUD to conduct proper test to determine its suitability* (Female, 28 years, married, 2 children, family planning user, middle SES, Ibadan).

### 3.4 Absolute trust placed in health professionals, health system, and government for contraceptive safety

An absolute trust in health professionals, hospitals, and governments was evident from many of the study participants in regards to family planning method advice: *[Pill use] is not harmful since it*’*s at the hospital they gave her* (Female, 20 years, unmarried, 0 children, low SES, Ibadan). The government was identified by one married man from Kaduna as a trusted endorser of family planning methods: *Pill is the least risky. If it has any health hazards, the doctors and government would not have given permission for its use* (44 years, 6 children, middle SES).

The level of trust in doctors’ ability to choose a family planning method with no side effects was demonstrated by study participants: *It has no danger, because the doctor that gives the injection knows it doesn*’*t have any side effects* (Male, married, 1 child, low SES, Kaduna). *In my own opinion, it is very good because it is a doctor that prescribed the drug, and I know that he cannot give drugs that will harm his patient* (Male, 24 years, married, 0 children, low SES, Kaduna). *It has no danger, because the doctor that gives the injection knows it doesn*’*t have any side effects* (Male, 23 years, married, 1 child, low SES, Kaduna).

In some instances, the mere availability of a particular family planning method was perceived as an endorsement that the method would be free from risk, as described by a married mother of two: *I think the oral pill is a good one for her because if it wasn*’*t good, it would not have been introduced to her or to any other woman in the first place* (35 years, family planning nonuser, low SES, Ibadan).

## 4 Discussion and conclusion

### 4.1 Discussion

This study aimed to understand contraceptive method choice from the perspective of the client in two cities in Nigeria. Study participants presented method choice as a medical decision—the active person making the decision was the health provider, most often referred to as the “doctor”. Other research in the Nigerian context has reported on how women indicate the doctor as a “very important” person in the decision-making surrounding contraceptive use [27].

The doctor uses the tools available, such as blood tests, to determine what contraceptive method would be best suited for the client, who was viewed as the passive recipient in this exchange. If a person used a contraceptive method that did not match with her body system/chemistry, then she would most certainly experience side effects from that method. Through the explanations of contraceptive method choice, an absolute trust in the health providers, the health system, and the Government of Nigeria to provide safe contraception without side effects was evident. This trust is important given that health workers are the second most common source of information on family planning methods [1].

### 4.2 Conclusion

While the trust of the health system, health professionals, and the Nigerian Government is among the most positive themes in regards to family planning use reported in this region—it is a bit disconcerting as the level of trust is so high that individuals might feel betrayed should anything go wrong when using family planning. This level of trust in the health provider is tenuous because a health provider cannot predict when a woman will experience side effects with method use or not—and most likely she will experience some side effects.

Studies conducted in Nigeria among college students, married women, and urban women show the most common reason for contraceptive nonuse is fear of side effects [5], [8], [20], [28]. Although fear of side effects is an important barrier to family planning use, only 47 percent of women in Nigeria using a modern method of contraception obtained from the private medical sector were informed about side effects or method-related problems, compared to 76 percent of women who used a public facility [1]. This is a concern because the majority of users of a modern method obtained their method from the private medical sector (60%), mostly from a patent medical store (PMS) (38%) [1]. These providers are not required to undergo professional training and may share inaccurate information – or no information at all – with their clients, and as demonstrated here, might be seen as trustworthy providers of family planning services.

Screening through tests and client histories can be helpful tools to identify contraindications to particular contraceptive methods but these tools are not able to predict side effects. The potential for side effects can only be identified through actual use of a method. The best solution to a client’s experience of unacceptable side effects is to switch to another contraceptive method. Switching methods is quite common and the ability to do so is essential for client satisfaction and continued use [11]. It has been suggested that choice *and* change should form the basis of initial family planning counseling, emphasizing to clients that their first choice can be changed if it is found to be unsuitable or the needs of the client change over time [11]. Family planning programs can produce user-friendly messages that effectively communicate the potential need for method switching based on user-experience and empower family planning users to negotiate method switching with health care providers.

The expectation of provider-driven method choice is indicative of an external locus of control (LOC) for family planning decision-making among Nigerian men and women. Such an external LOC, as articulated in Rotter’s Social Learning Theory, means that clients believe that their outcomes are determined by external factors such as chance or powerful others rather than as a consequence of their own behaviors (internal LOC) [29]. An external LOC, especially related to powerful others, has been shown to influence health behaviors, such as increased HIV risk among immigrant women in the US [30] and lower use of contraceptives among adolescents in Brazil [31]. Research on older Nigerian women found that modern contraceptive use increased with increased decision-making power within the household [32]. In order to improve client involvement in family planning decision-making and method choice, family planning programs in Nigeria should consider approaches to increase internal LOC among potential and continuing family planning clients as part of their intervention.

This study suffers from a few limitations. The study was qualitative and conducted in just two urban areas in Nigeria—so the findings are not generalizable to the entire urban Nigeria population. Only one researcher coded the data. The groups were disaggregated by many factors, as a result it was difficult to find patterns across the demographic factors. Finally, study participants were not probed to discern whether contraceptive decision-making, and method choice, differed by type of provider or facility. Despite the limitations, there is a strength to this study. The study included projective techniques to encourage dialogue that might not have occurred with more standard style questioning.

Future research in this area might expand on the findings presented here – to discern whether contraceptive method decision-making differs by type of provider and type of facility, to determine what specific types of tests the clients perceive the provider to conduct on clients to determine body type – the specific purpose of each type of test and the meaning of particular test outcomes, the reaction by family planning users who have undergone the “testing” when they do suffer from side effects associated with contraceptive method use, and the effects of programs aiming to encourage prospective, and unsatisfied, family planning clients to take charge of family planning decision making.

### 4.3 Practical implications

Through this study a number of issues emerged surrounding contraceptive method decision-making that could inform development of messaging and policy changes. First, communication campaigns could work to de-mystify the process that health professionals use to support contraceptive decision-making. Communication campaigns should also help clients understand their important role in method choice by increasing their internal locus of control about contraceptive method decision-making. These campaigns would work best if done in tandem with training among contraceptive providers on client-centered counseling, including the important role of clients in the selection of the contraceptive method.

Second, given how important switching contraceptive methods is in response to unmanageable side effects, contraceptive providers should be trained to discuss the strategy of switching to all clients—potential future clients, new clients, and continuing clients. Third, all persons who provide contraceptive methods, including those in the private sector, would benefit from training on client-centered counseling, especially related to counseling all clients – new and returning – on potential side effects.

Widely disseminating accurate information about the importance of individual preference in contraceptive method choice, and the ability to switch methods, could increase contraceptive use in Nigeria through increased use among non-users, satisfaction with use among current users, and the power that comes from feeling in control.

## Figures and Tables

**Table 1:** Focus group discussions by attribute, Ibadan and Kaduna, Nigeria, 2010.

City	Sex	Age	Marital Status	Neighborhood Socioeconomic Status	Family Planning Use
Ibadan	Female	18–24 years	Not Married	Low	
Married	Low	Never
Current
Middle	Never
Current
25–49 years	Married	Low	Never
Current
Middle	Never
Current
Male	18–24 years	Not Married	Low	
Married	Low	
Middle	
25–49 years	Married	Low	
Middle	
Kaduna	Female	18–24 years	Married	Low	Never
Current
Middle	Never
25–49 years	Married	Low	Never
Current
Middle	Never
Current
Male	18–24 years	Not Married	Low	
Married	Low	
Middle	
25–49 years	Married	Low	
Middle	

## References

[1] National Population Commission (NPC) [Nigeria] (2014). ICF International. Nigeria Demographic and Health Survey 2013.

[2] Cleland J.G., Ndugwa R.P., Zulu E.M. (2011). Family planning in sub-Saharan Africa: progress or stagnation. Bull. World Health Org..

[3] Monjok E., Smesny A., Ekabua J.E. (2010). Contraceptive practices in Nigeria: literature review and recommendation for future policy decisions. J. Contracept..

[4] Williamson L.M., Parkes A., Wight D., Petticrew M., Hart G.J. (2009). Limits to modern contraceptive use among young women in developing countries: a systematic review of qualitative research. Reprod. Health.

[5] Obisesan K.A., Adeyemo A.A., Fakokunde B.O. (1998). Awareness and use of family planning methods among married women in Ibadan, Nigeria. East Afr. Med. J..

[6] Abiodun O.M., Balogun O.R. (2009). Sexual activity and contraceptive use among young female students of tertiary educational institutions in Ilorin, Nigeria. Contraception.

[7] Omideyi A.K., Akinyemi A.I., Aina O.I., Adeyemi A.B., Fadeyibi O.A., Bamiwuye S.O. (2011). Contraceptive practice, unwanted pregnancies and induced abortion in southwest Nigeria. Glob. Public Health.

[8] Adeyinka D.A., Oladimeji O., Adeyinka E.F., Adekanbi I.T., Falope Y., Aimakhu C. (2009). Contraceptive knowledge and practice: a survey of under graduates in Ibadan, Nigeria. Int. J. Adolesc. Med. Health.

[9] Ugboaja J.O., Nwosu B.O., Ifeadike C.O., Nnebue C.C., Obi-Nwosu A.I. (2011). Contraceptive choices and practices among urban women in southeastern Nigeria. Niger. J. Med..

[10] Sullivan T.M., Bertrand J.T., Rice J., Shelton J.D. (2006). Skewed contraceptive method mix: why it happens why it matters. J. Biosoc. Sci..

[11] Bruce J. (1990). Fundamental elements of the quality of care: a simple framework. Stud. Fam. Plan..

[12] Hamid S., Stephenson R. (2006). Provider and health facility influences on contraceptive adoption in urban Pakistan. Int. Fam. Plan. Perspect..

[13] Pariani S., Heer D.M., Van Arsdol M.D. (1991). Does choice make a difference to contraceptive use? Evidence from East Java. Stud. Fam. Plan..

[14] Bulatao R.A., Bulatao R.A., Palmore J.A., Ward S.E. (1989). Toward a framework for understanding contraceptive method choice. Choosing a Contraceptive: Method Choice in Asia and the United States.

[15] Gubhaju B. (2009). The influence of wives’ and husbands’ education levels on contraceptive method choice in Nepal, 1996–2006. Int. Perspect. Sex. Reprod. Health.

[16] Magadi M.A., Curtis S.L. (2003). Trends and determinants of contraceptive method choice in Kenya. Stud. Fam. Plan..

[17] Mannan H.R. (2002). Factors in contraceptive method choice in Bangladesh: goals, competence, evaluation and access. Contraception.

[18] Stephenson R., Beke A., Tshibangu D. (2008). Community and health facility influences on contraceptive method choice in the Eastern Cape, South Africa. Int. Fam. Plan. Perspect..

[19] Hebert L.E., Schwandt H.M., Boulay M., Skinner J. (2013). Family planning providers’ perspectives on family planning service delivery in Ibadan and Kaduna, Nigeria: a qualitative study. J. Fam. Plan. Reprod. Health Care.

[20] Black T. (1999). Impediments to effective fertility reduction. Contraception should be moved out of the hands of doctors. BMJ.

[21] Grossman D., Ellertson C., Abuabara K., Blanchard K., Rivas F.T. (2006). Barriers to contraceptive use in product labeling and practice guidelines. Am. J. Public Health.

[22] Shelton J.D., Angle M.A., Jacobstein R.A. (1992). Medical barriers to access to family planning. Lancet.

[23] WHO (2004). Selected Practice Recommendations for Contraceptive Use. http://www.who.int/reproductivehealth/publications/family_planning/9241562846index/en/.

[24] Federal Ministry of Health Nigeria (2009). National Family Planning/Reproductive Health Service Protocols. http://www.nurhitoolkit.org/sites/default/files/tracked_files/National%20Service%20Protocols%20for%20FP.pdf.

[25] Green J., Thorogood N. (2004). Qualitative Methods for Health Research, USA.

[26] Keenan K.F., van Teijlingen E., Pitchforth E. (2005). The analysis of qualitative research data in family planning and reproductive health care. J. Fam. Plan. Reprod. Health Care.

[27] Orubuloye I.O., Oguntimehin F., Sadiq T. (1997). Women’s role in reproductive health decision making and vulnerability to STD and HIV/AIDS in Ekiti, Nigeria. Health Transit Rev..

[28] Arowojulu A.O., Ilesanmi A.O., Roberts A.O., Okunola M.A. (2002). Sexuality, contraceptive choice and AIDS awareness among Nigerian undergraduates. Afr. J. Reprod. Health.

[29] Rotter J.B. (1966). Generalized expectancies of internal versus external control of reinforcements. Psychol. Monogr..

[30] Loue S., Cooper M., Traore F., Fiedler J. (2004). Locus of control and HIV risk among a sample of Mexican and Puerto Rican women. J. Immigr. Health.

[31] Alves A.S., Lopes M.H. (2010). Locus of control and contraceptive knowledge, attitude and practice among university students. Rev. Saúde Públ..

[32] OlaOlorun F.M., Hindin M.J. (2014). Having a say matters: influence of decision-making power on contraceptive use among Nigerian women ages 35–49 years. PLoS One.

